# Effect of Isothermal Transformation Times below Ms and Tempering on Strength and Toughness of Low-Temperature Bainite in 0.53 C Bainitic Steel

**DOI:** 10.3390/ma13102418

**Published:** 2020-05-25

**Authors:** Enzuo Liu, Qiangguo Li, Sufyan Naseem, Xuefei Huang, Weigang Huang

**Affiliations:** 1 College of Materials Science and Engineering, Sichuan University, Chengdu 610065, China; Liu_Enzuo@163.com (E.L.); sfyn24@stu.scu.edu.cn (S.N.); huangxf08@scu.edu.cn (X.H.); 2 College of Architecture and Environment, Sichuan University, Chengdu 610065, China; LeeValiant@163.com

**Keywords:** low-temperature bainite, martensite, carbide free bainite, isothermal treatment, impact toughness

## Abstract

This study aims to investigate the microstructures, strength, and impact toughness of low-temperature bainite obtained by isothermal transformation at temperature below Ms (Martensite Starting temperature) for different times and tempering process in 0.53 C wt% bainitic steel. By using the optical microscopy, X-ray diffraction (XRD), transmission electron microscopy (TEM), electron back scatter diffraction (EBSD), and mechanical property test, it was found that the microstructures after heat treatment consist of small amounts of martensite, fine bainite, and film retained austenite. After tempered at 250 °C for 2 h, the volume fraction of retained austenite (10.9%) in the sample treated by isothermal transformation at 220 °C for three hours is almost the same as that of the sample without tempering. In addition, the retained austenite fraction decreases with the increase of holding times and is reduced to 6.8% after holding for 15 h. The ultimate tensile strength (1827 MPa), yield strength (1496 MPa), total elongations (16.1%), and impact toughness (up to 58 J/cm^2^) were obtained by isothermal transformation at 220 °C for three hours and tempered at 250 °C. Whereas, the impact toughness of sample without tempering is 28 J/cm^2^. After holding for 15 h, the impact toughness raises to 56 J/cm^2^, while the ductility and strength decreases. These results indicate that the tempering process is helpful to improve the impact toughness of low-temperature bainite.

## 1. Introduction

Recently, carbide-free bainite has attracted more attention from researchers for its high strength and good toughness. Carbide-free bainitic steels, as promising advanced, high-strength steels, are believed to have many applications in industries, such as automobile [1], rail [2,3,4], railway crossing [5,6], and bearing [7,8]. Previous research has demonstrated that the excellent strength and toughness of carbide-free bainite can be attributed to the fine bainitic ferrite size and the thin films of retained austenite between lath or the subunits of bainitic ferrite [9]. The thin film retained austenite with higher carbon content is more stable than blocky retained austenite, which can suppress the micro cracks initiation and retard the propagation of crack in carbide-free bainite steel [10,11]. In addition, the improvement of ductility and the toughness in carbide-free bainitic steel can be attributed to TRIP (Transformation Induced Plasticity) effect of uniform distribution of film retained austenite [1,5,12,13,14].

Based on the concept of carbide-free bainite, low-temperature bainite obtained by isothermal transformation of austenite at low-temperature in the high carbon high silicon steel was investigated firstly by Sandivik and Nevalainen [15]. They indicated that the low-temperature bainite in high carbon high silicon steel with high strength and ductility consists of ultra-fine bainitic ferrite and film retained austenite. Then, further investigations about low-temperature bainite were carried out by Bhadeshia’s group [16,17,18,19]. Their work showed that the very fine bainite and film dispersion of retained austenite between the bainitic ferrite can be obtained in the high carbon, high silicon steel by isothermal transformation at temperature as low as *T* ≅ 0.25 *Tm* (Tm: absolute melting temperature). The very fine low-temperature bainite has an ultimate high tensile strength of over 2.3 GPa and a good toughness of 30 MPam^1/2^. It was found that the addition of Al and Co in steel can accelerate the low-temperature bainite transformation [19]. Garcia-Mateo and Caballero et al. [12] discovered that the low-temperature bainite in high carbon steel with ~3 Si wt%, instead of the expensive Co element, exhibits an unrivalled combination of ultimate tensile strength (above 2 GPa) and ductility (above 21% total elongation). Zhang et al. [20] reported on the microstructure and properties of low-temperature bainite obtained in a high carbon Si-Al-rich steel by adding Al to accelerate the bainite transformation. The microstructure consists of bainite ferrite plates of 38–57 nm and film retained austenite. The nanosized bainite microstructure exhibits the yield strength of 1534–1955 MPa, ultimate tensile strength of 2080–2375 MPa, and the impact toughness of 7.8–22.2 J. As reported above, studies on low-temperature bainite were carried out predominantly in high carbon alloy steels. High carbon concentration is a necessary requirement to decrease the Ms and Bs temperature so as to allow bainite transformation to be carried out at low temperature [16]. In order to avoid the poor weldability of high carbon steels, many works on the low-temperature bainite with low or medium carbon bainite steel has been studied by many researches. Yang and Bhadeshia [21] found that high Ni concentration in 0.2 wt% C steel indeed suppressed the Ms temperature but it can coalesce the bainite ferrite. Soliman et al. [22] reported that the low-temperature bainite can be obtained in steel with 0.26 wt% C due to the effective suppression of the Ms temperature by addition of 1.85 wt% Ni and 3.44 wt% Mn, and the fine low-temperature bainite exhibits ultimate compression strength of 2.2 GPa. 

Recently, it has been reported that the low-temperature bainite with fine bainitic ferrite and thin films of retained austenite can be obtained below Ms temperature in the low and medium carbon alloy steels and exhibit higher impact toughness and tensile strength than that of bainite obtained above Ms temperature [23,24,25]. The reason for good mechanical properties is that for low and medium carbon steels, the bainite transformation below Ms temperature can reduce or even eliminate the existence of blocky retained austenite and ensure that the microstructure consists of the fine bainitic ferrite plates and film retained austenite. Wang et al. [26] proposed a multi-step low-temperature isothermal bainite transformation below Ms temperature to obtain the fine bainitic ferrite and film retained austenite. This heat treatment effectively improves the strength and impact toughness of tested steel. Avishan et al. [27] carried out the two-step isothermal transformation at low temperature in high carbon steel to obtain the nanosized bainitic ferrite and film retained austenite. The result indicated that the two-step isothermal treatment process can enhance the strength, ductility, and impact toughness of high carbon steels. Many other researches on the characteristics of bainite transformation below Ms temperature, including microstructure, kinetics, crystallography, etc., have been reported by previous authors [28,29,30,31,32,33].

Based on the previous works stated above, in this research work, the 0.53 wt% C bainite steel with Al was used to mainly investigate effects of the different isothermal holding times below Ms temperature and tempering process on the microstructure and mechanical properties of low-temperature bainite. The main purpose of this work is to improve the toughness of low-temperature bainite in medium carbon steel while maintaining its high strength. 

## 2. Experimental Material and Procedures

The chemical composition of the designed medium-carbon alloyed steel in wt% was Fe-0.53C-1.5Mn-1.6Si-1.5Cr-1.3Al-0.3Mo-0.05Nb. Si was added to suppress carbide precipitation during the bainite transformation and tempering process. Mn and Cr can improve the hardenability and decrease the transformation temperature of bainite and martensite. Al has an effect on accelerating bainite transformation at low temperatures. Mo can delay the transformation of proeutectic ferrite and pearlite in high temperature. Nb can refine austenite grain during high temperature deformation and is beneficial for improving the strength and toughness of steel.

**Figure 1:**
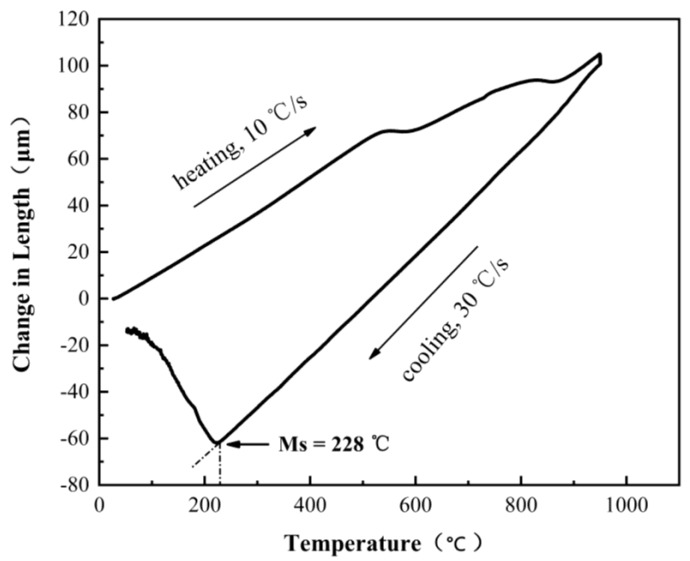
The dilatation curve of experimental steel.

The experimental steel was melted in a 30 kg vacuum induction furnace (JKZ, Chengdu, China). The casting ingot was forged at about 1200 °C into a billet with 65 mm width and 25 mm thickness. The forged billets were cooled in air after forging. The Ms temperature was tested to be 228 °C by Bahr DIL805 A/D dilatometer (TA Instruments, New Castle, DE, USA). The solid cylindrical sample with 4 mm in diameter and 10 mm in length was used for measurement of Ms temperature. During the test process, the sample was first heated to the austenite temperature of 950 °C at a heating rate of 10 °C/s and held there for 10 min in the vacuum chamber. After that, it was cooled to room temperature at a cooling rate of 30 °C/s by blowing pure helium gas. The dilatation curve of experimental steel is shown in Figure 1. 

**Figure 2:**
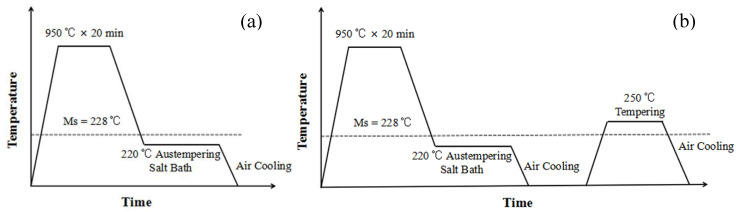
Heat treatment processes of low-temperature bainite transformation in the experiment. (**a**) isothermal below Ms (**b**) isothermal and tempering.

The heat treatment processes adopted in this study are shown in Figure 2. Before isothermal heat treatment, all samples with the shape and the size with machining allowance for tensile and impact toughness test were cut from the forged steel and annealed at 900 °C for 30 min. For the bainite isothermal process below Ms temperature, as shown in Figure 2a, the samples were austenitized at 950 °C for 20 min and then immediately placed into salt bath with isothermal holding at 220 °C for different times to obtain different amounts of low-temperature bainite. The heat treatment of samples was carried out by using a resistant furnace (Boxun, Shanghai, China), in which some charcoals were put to prevent samples from decarburization and oxidation. To investigate the effect of tempering on the strength and toughness of low temperature bainite, the heat treatment process of the isothermal transformation below Ms temperature and tempering at 250 °C for 2 h were proposed, as shown in Figure 2b.

Tensile property tests were performed on an electronic material testing machine (DDL100, Sinotest Equipment Co, Changchun, China) at a strain rate of 3 × 10^−3^ s^−1^. Tensile specimens with a gauge diameter of 5 mm and a gauge length of 23 mm were used and three samples were tested for each heat treatment process to measure the strength and ductility of the experimental samples. Standard Charpy U-notched impact samples with a size of 10 × 10 × 55 mm were used for an impact toughness test on an impact tester (JB-300B, Shengliang, Jinan, China). Three specimens were tested for each treatment. The hardness of all samples was measured by digital display Rockwell Hardness Tester (HR-150, SCTMC, Shanghai, China) with a load of 150 kg. The hardness value (HRC) was obtained by the average of five indentation values. All of the mechanical property tests were carried out at room temperature.

The microstructure characterization of samples treated by different heat treatment process was observed by optical microscopy (MDS400, Cnoptec, Chongqing, China) and scanning electron microscopy (SEM, Apreo S, Thermo Scientific, Waltham, MA, USA) after the samples were mechanically groud, polished, and then etched with 4% nital solution. The samples for transmission electron microscope (TEM; JEM-2010, JEOL Ltd., Tokyo, Japan) analysis were polished to 50 μm thickness and then electropolished with an electrolyte of 4% perchloric acid and 96% ethanol at −20 °C. The electron backscatter diffraction (EBSD) examination was carried out in a field emission scanning electron microscope (FE-SEM; JSM 7001F, JEOL Ltd., Tokyo, Japan) equipped with EBSD system (EDAX/TSL) with accelerating voltage of 20 kV, working distance of 15 mm, tilt angle of 70° and step size of 150 nm. The EBSD data analysis was performed with TSL-OIM Analysis 7.1 software (EDAX, San Diego, VA, USA). The volume fraction of retained austenite was determined by DX-2700 X-ray diffractometer (XRD, Shjingmi, Shanghai, China) with CuK_α_ radiation, 40 kV, 30 mA at room temperature and calculated using the diffraction intensity of (211)_α_ and (220)_γ_, according to the equation as follows (1):

(1) $$V_{RA}=\frac{1}{1+0.65\times \frac{I_{\left(211\right)\alpha }}{I_{\left(220\right)\gamma }}}$$

## 3. Results and Discussion

### 3.1. Microstructure Characterization

**Figure 3:**
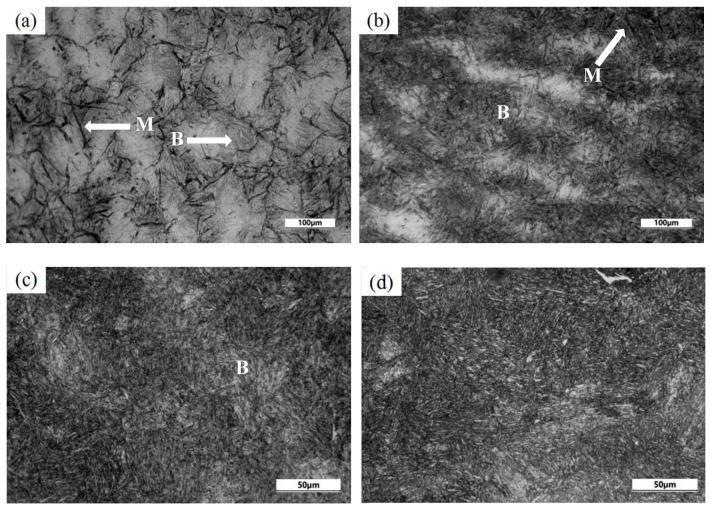
Optical micrographs of experimental steel treated by isothermal transformation at 220 °C for different times: (**a**) 2 h; (**b**) 3 h; (**c**) 7 h; (**d**) 15 h. (M: martensite, B: bainite).

The low-temperature bainite transformation was carried out at 220 °C, which is below the Ms temperature of 228 °C. Figure 3 shows the optical micrographs of samples treated by isothermal transformation at 220 °C for 2 h, 3 h, 7 h, and 15 h. As shown in Figure 3a, the microstructure obtained by isothermal transformation at 220 °C for 2 h consists of martensite with black contrast and thin acicular bainite with slight contrast within grain, which is consistent with the results reported by Yuki Toji [34]. According to the equation [35]:

(2) $$f_{m}=1-e^{-1.1\times 10^{-2}\left(M_{s}-QT\right)}$$

where *f_m_* represents the fraction of martensite obtained by quenching to a temperature between Ms and Mf. QT is the quenching temperature below Ms temperature. Therefore, the volume fraction of martensite was estimated to be about 8.4% by first quenching at 220 °C before isothermal bainite transformation. Then, a lot of thin bainite plates were formed by isothermal holding for 2 h, as shown in Figure 3a. This indicates that the experimental steel exhibits the fast transformation behavior of bainite during isothermal transformation at low temperature below Ms. It is estimated that the volume fraction of bainite is about 90% when the isothermal holding times increase to 3 h, as shown in Figure 3b. As seen from the Figure 3c,d, the volume of bainite in samples increases with the increase of holding time and the thin acicular bainitic ferrite becomes thick as the isothermal holding time increase to 7 h and 15 h.

**Figure 4:**
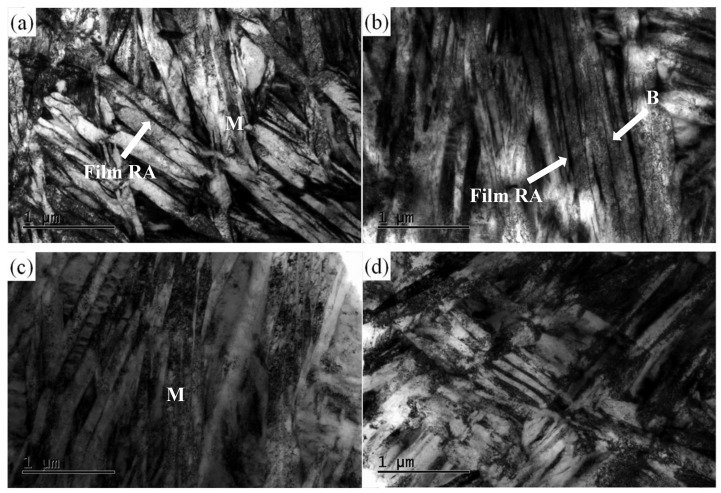
TEM images of experimental steel treated by isothermal transformation at 220 °C for different times and tempering: (**a**) 3 h; (**b**) 3 h and tempered at 250 °C for 2 h; (**c**) 7 h; (**d**) 15 h. (M: martensite, B: bainite, Film RA: film retained austenite).

Figure 4 shows the TEM micrographs of tested samples under different heat treatment processes. The microstructure obtained by isothermal transformation at 220 °C for 3 h and tempered at 250 °C for 2 h consists of lath martensite, bainite, and retained austenite, as shown in Figure 4a,b. Figure 4a demonstrates the lath martensite and thin film retained austenite between martensitic laths with high density of dislocation and no existence of carbides in martensite laths. Figure 4b exhibits the bainitic ferrite and the thin films of retained austenite. However, it is clear that the bainite ferrite plates are short in length and slightly wide in size compared with martensite shown in Figure 4a. These bainite ferrite with small size formed below Ms for the tested steel may be attributed to the following major reasons. Firstly, the effect of geometrical partitioning of prior austenite grain by the formation of prior martensite lathes and bainite ferrite on the refinement of untransformed austenite, which reduce the space of bainite growing. Secondly, the high dislocation density in untransformed austenite introduced by prior martensite is believed to be beneficial for bainite transformation at low temperature. Finally, many phase interfaces between martensite and untransformed austenite produced by prior martensite formation may be responsible for the refinement of bainite microstructure [34,36]. The retained austenite in the bainite microstructure consists of film-like and blocky retained austenite, as shown in the Figure 4b. Figure 4c,d shows the microstructure of samples for isothermal transformation at 220 °C for 7 and 15 h, respectively. The microstructure clearly indicates the lath bainitic ferrite and film retained austenite. The bainitic ferrite laths are still refined for a long isothermal holding times and no carbides were found in the bainitic ferrites, which can be attributed to the positive effect of Si and Al elements in the steel on the inhibition of the carbide precipitation during the transformation of martensite and bainite as well as the tempering process at 250 °C.

**Figure 5:**
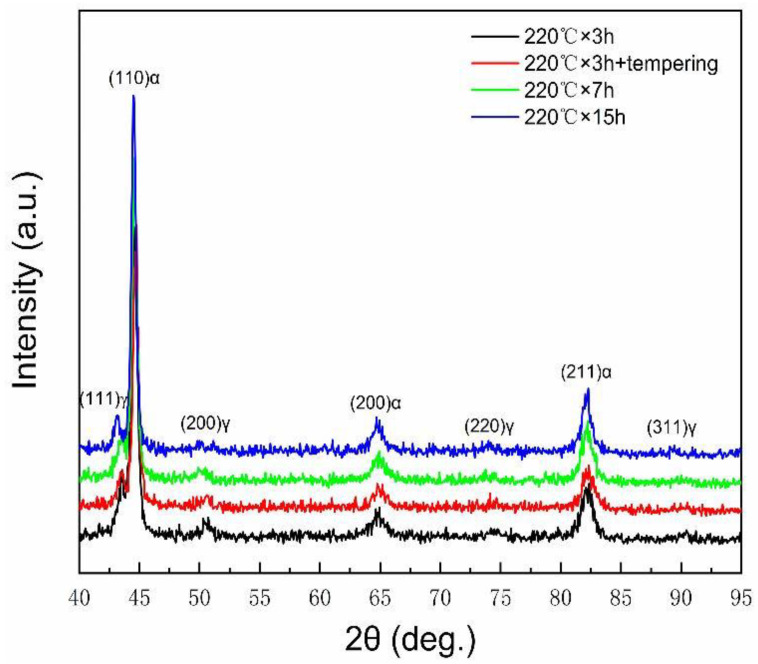
XRD patterns of the experimental steel after different heat treatment process.

Figure 5 shows the X-ray diffraction (XRD) patterns of the samples subjected to different heat treatment process and the calculated volume fractions of retained austenite are listed in Table 1. As seen from Figure 5, only ferrite (α) and austenite (γ) phase are present in all samples and no carbides phases are observed. The volume fractions of retained austenite for samples after isothermal heat treatment for 3 h with or without tempered at 250 °C for 2 h are 10.9% and 10.6%, respectively, as shown in the Table 1. The results indicate that there is no obvious effect of tempering at 250 °C on the volume fractions of retained austenite. It can be considered that the retained austenite in the samples exbibits good stability by isothermal process at 220 °C for 3 h. However, the volume fraction of retained austenite decreases to 9.5% and 6.8% at the isothermal holding for 7 and 15 h, respectively, as shown in Table 1. It may be responsible for the further transformation of bainite at the isothermal temperature for a long time holding continuously.

### 3.2. Mechanical Properties

The mechanical properties of the experimental steel treated by different heat treatment processes were investigated and the results are summarized in Table 2. The hardness of the samples treated by isothermal transformation at 220 °C for 3 h without or with tempering is 55.1 and 55.6 HRC, respectively. With the increase of isothermal holding times, the hardness slightly decreases to 54.3 and 53.3 HRC after prolonging the holding times to 7 h and 15 h, respectively. As seen from Table 2, the tensile strength of the samples after isothermal transformation at 220 °C for 3 h and tempered at 250 °C for 2 h shows the highest ultimate tensile strength (1827 MPa) and yield strength (1496 MPa). The uniform and total elongations are 10.5% and 16.1%, respectively. The ultimate tensile strength and yield strength decrease to 1741 MPa, 1357 MPa, and 1797 MPa, 1578 MPa for the sample with isothermal transformation at 220 °C for 7 h and for 15 h, respectively. The uniform and the total elongation of samples holding for 7 h are 11.9% and 16.2%. This shows that the ductility exhibits no obvious difference compared with the sample subjected to isothermal transformation at 220 °C for 3 h and tempering process. However, for the sample holding for 15 h, the uniform and total elongation decease to 9.1% and 12.9%.

**Figure 6:**
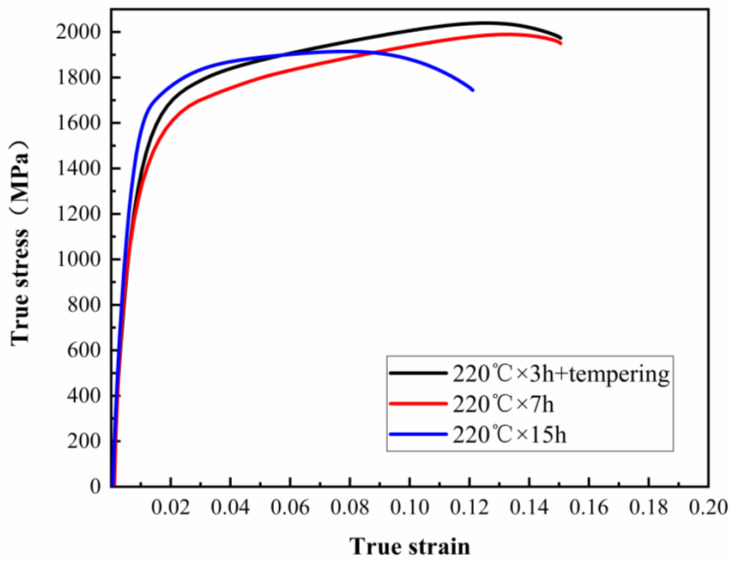
The true stress-strain curves of test steel by different heat treatment processes.

The true stress-strain curves of samples with different heat treatment processes are shown in Figure 6. As seen from Figure 6, the continuous yielding behaviors of the tested samples are observed in the true stress-strain curves, which can be related to the existence of more mobile dislocations within the microstructure [12]. It clearly shows that the samples of isothermal transformation at 220 °C for 3 h and tempered at 250 °C for 2 h and at 220 °C for 7 h exhibit an obvious plateaus of strain hardening before necking, which indicates that the samples demonstrate obvious uniform deformation performance, leading to good ductility. However, the sample held for 15 h at 220 °C shows low ability of uniform deformation. The true stress-strain behaviors of steel can be described by Hollomon equation:

(3) $$\sigma =K\varepsilon^{n}$$

where σ and ε represent true stress and true strain, *K* is a constant, and n refers to strain hardening exponent. From Equation (3), the logarithmic equation can be derived, as follows:

(4) lnσ=lnK+nlnε

According to the Equation (4), the lnσ−lnε curves were plotted. Then, the strain hardening exponent, n, can be obtained by the following equation in the uniform deformation range:

(5) n=d(lnσ)/d(lnε)

According to the calculated results, the strain hardening exponent, n, of samples treated by different heat treatment processes are 0.103, 0.135, and 0.072, respectively. It shows that the samples subjected to isothermal transformation for 3 h and the tempering process and for 7 h at 220 °C have higher uniform strain performance than that of the sample held for 15 h at 220 °C. This result can be attributed to the more volume fraction of film retained austenite in the microstructure. As seen from Figure 4, the film retained austenite is located between the laths of martensite or bainitic ferrite. This kind of film retained austenite exhibits high stability due to subjected to high compressive stress caused by the surrounding martensite or bainite ferritic laths. The highly stable film retained austenite can contribute to the inhibition of the crack initiation in the microstructure, thus effectively improve the TRIP effect under the uniform deformation [12,37]. However, it is found that although the volume fraction of retained austenite of the sample with isothermal transformation at 220 °C for 7 h is slightly lower than that of the sample with isothermal transformation at 220 °C for 3 h and tempered at 250 °C, the uniform strain and strain hardening exponent are higher. It may be attributed to the increase in the amount of bainite microstructure obtained by increasing the holding time. As a result, owing to the increase of bainite, the strength mismatch between the bainitic ferrite and retained austenite is considered to be moderate, which can relieve the stress concentration within different phases [38,39]. The low uniform strain and strain hardening exponent of the sample processed by holding for 15 h at 220 °C should be attributed to the low volume fraction of retained austenite, resulting in decreasing the TRIP effect.

The impact toughness test was carried out to investigate the toughness of samples with different heat treatment processes. The results are listed in Table 3. As seen from Table 3, the sample treated by isothermal transformation at 220 °C for 3 h and tempered at 250 °C has a high impact toughness of 58 J/cm^2^, whereas the impact toughness of sample that is not tempered is 28 J/cm^2^. The low impact toughness can be attributed to the existence of fresh martensite with carbon supersaturation and the lattice distortion resulted from the supersaturated carbon in martensite because the microstructure was subjected to tempering. Therefore, the significant improvement of impact toughness of the sample with tempering can be attributed to higher stability of retained austenite and the residual stress relief in the sample due to further carbon partitioning from the martensite to retained austenite and decrease in lattice distortion after tempered at 250 °C [40]. It can be seen from Table 3 that the impact toughness of samples increases with increase of isothermal holding time at 220 °C, the values of which are 44 J/cm^2^ and 56 J/cm^2^ after treated by isothermal holding time for 7 h and 15 h at 220 °C, respectively. Although the impact toughness can be improved under a long time holding, it is still less than that of the sample obtained by isothermal transformation at 220 °C for 3 h and tempered at 250 °C. The increase of impact toughness with increase of isothermal holding time may be attributed to the following reasons. Firstly, the more bainite microstructure can be obtained due to extension of the isothermal transformation time and the volume of brittle fresh martensite produced during air cooling to room temperature after holding for a long isothermal time will be reduced or even eliminated. Secondly, the internal stress within lattice can be released as much as possible because the carbon in martensite formed by initial quenching at 220 °C and low-temperature bainite ferrite can be partitioned into the untransformed austenite during a long isothermal holding time, leading to improving the stability of retained austenite [41]. Finally, the volume fraction of blocky retained austenite can be decreased. Therefore, although the total volume fraction of retained austenite decreases, the stable film retained austenite is beneficial to improve the impact toughness [24,25,26,27,42]. The key role of more stable film retained austenite in improving the impact toughness can be contributed to retarding the formation of microcracks, effectively blunting the crack tip as well as causing more energy consumption during the crack propagation process mainly by the TRIP effect [43,44].

**Figure 7:**
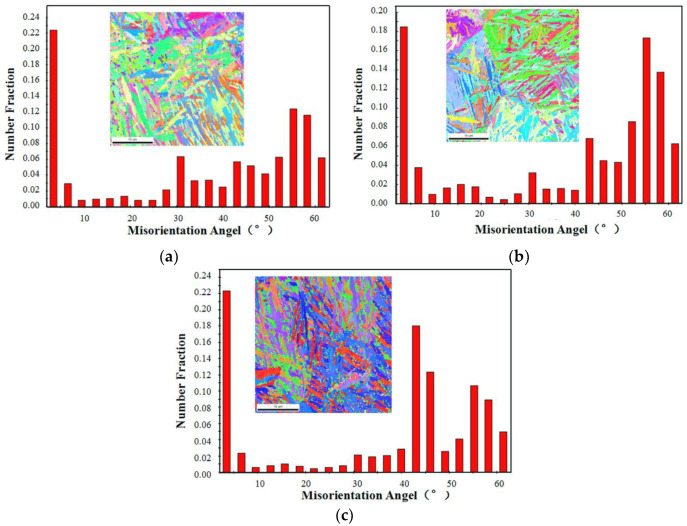
The boundary misorientation angle statistical column graphs of the experimental steel after different isothermal process. (**a**) 220 °C × 3 h + tempering, (**b**) 220 °C × 7 h, and (**c**) 220 °C × 15 h.

It can be seen from the heat treatment process described above that all samples were heat treated under the same heating and isothermal temperature. Therefore, it can be inferred that the microstructure size in samples with different heat treatment process, such as austenite grain, bainite ferritic lathes, etc., are almost same as each other. Figure 7 shows the boundary misorientation angle distribution maps of different heat treatment processes. As seen from Figure 7, the proportion of boundary misorientation angles greater than 15° is about 74% in samples for all three heat treatment processes, which shows no significant difference. The boundary misorientations angles greater than 15° are considered high angle boundaries, which should involve austenite grain boundaries, packet boundaries, and block boundaries in the lath-like microstructure, such as martensite and bainite [37,45,46,47]. These high angle boundaries in the microstructure can act as a barrier of crack propagation, leading to more energy consumption during the fracture process. The boundary misorientation lower than 15° is considered to be a low angle boundary, which corresponds to lath boundaries of martensite or bainite [46,47]. As reported by Yilong Liang and Shao-lei Long [47,48], the fracture toughness improves with the increase of the number fraction of low angle boundaries that are lower than 10°. According to the EBSD results in Figure 7, the proportion of boundary misorientation angle lower than 15° is about 27%, 25%, and 26% for the process of isothermal transformation at 220 °C for 3 h and tempering, isothermal transformation at 220 °C for 7 h and isothermal transformation at 220 °C for 15 h, respectively. The 27% high proportion of boundary misorientation lower than 15° may take a role in the improvement of impact toughness of the sample subjected to the isothermal process at 220 °C for 3 h and tempering.

From the results discussed above, it can be believed that the improvement of ductility and impact toughness of samples are probably related to the stable film retained austenite and residual stress relief in martensite and low-temperature bainite Increasing isothermal holding times can increase the volume fraction of low-temperature bainite and the stability of the film retained austenite, resulting in the increase of impact toughness although the volume fraction of retained austenite decreases.

## 4. Conclusions

Fe-0.53C-1.5Mn-1.7Si-1.5Cr-1.3Al-0.3Mo-0.05Nb bainitic steel was used to investigate the microstructure and mechanical properties by isothermal heat treatment process at a temperature below Ms for different holding times. The effects of tempering on the strength, ductility, and impact toughness of low-temperature bainite were also studied. The results are summarized as follows:

1. The microstructure consists of martensite, bainite, and retained austenite through isothermal transformation at 220 °C below Ms. The martensite contents are about 8.4% under initial quenching temperature of 220 °C. The amount of low-temperature bainite increases with the increase of isothermal transformation times. However, the size of bainitic ferrite can remain small under isothermal holding for a long time at 220 °C.
2. The retained austenite in samples mainly shows film-like morphology, located between bainitic ferrite laths. The volume fractions of retained austenite decreases from 10.6% to 6.8% with the increase of isothermal holding time. However, the retained austenite contents can be maintained at 10.9% after tempering at 250 °C for 2 h.
3. The samples after being treated at 220 °C for 3 h without and with tempering show 55.1 and 55.6 HRC, respectively. With the increase of isothermal holding times, the hardness slightly decreases to 54.3 and 53.3 HRC.
4. The sample after isothermal holding at 220 °C for 3 h and tempered at 250 °C for 2 h exhibits high ultimate tensile strength (1827 MPa), yield strength (1496 MPa), and impact toughness of 58 J/cm^2^. While the impact toughness of sample without tempered is 28 J/cm^2^, for the sample with isothermal transformation for 15 h at 220 °C, the toughness is 56 J/cm^2^; however, the uniform elongation decreases to 9.1%.

On the whole, the best combination of high strength and good toughness in the 0.5 C bainitic steel that can be obtained by the isothermal transformation at 220 °C below Ms plus tempering at 250 °C. It can be attributed to existence of multiphase microstructure with fine martensite, bainitic ferrite plates, appropriate amount of film retained austenite, and the sufficient release of residual stress.

## Figures and Tables

**Table 1:** The volume fractions of retained austenite in samples subjected to different heat treatment process (V_AR_%).

Heat Treatment Process	V_RA_ (%)
220 °C × 3 h	10.6
220 °C × 3 h + tempered	10.9
220 °C × 7 h	9.5
220 °C × 15 h	6.8

**Table 2:** The mechanical properties of the experimental steels underwent different heat treatment processes. (UEL: uniform elongation, TEL: total elongation, n: strain hardening exponent, YS: yield strength, UTS: ultimate tensile strength).

Heat Treatment Process	H (HRC)	YS (MPa)	UTS (MPa)	UEL (%)	TEL (%)	n
220 °C × 3 h + tempering	55.6 ± 0.36	1496 ± 23	1827 ± 27	10.5 ± 0.8	16.1 ± 1.9	0.103
220 °C × 7 h	54.3 ± 0.30	1357 ± 12	1741 ± 15	11.9 ± 0.7	16.2 ± 0.7	0.135
220 °C × 15 h	53.3 ± 0.26	1578 ± 31	1797 ± 39	9.1 ± 0.2	12.9 ± 1.2	0.072

**Table 3:** The impact properties of the experimental steels underwent different heat treatment processes.

Heat Treatment Process	a_KU_ (J/cm^2^)
220 °C × 3 h	28 ± 3
220 °C × 3 h + tempering	58 ± 1
220 °C × 7 h	44 ± 3
220 °C × 15 h	56 ± 1
